# Inhibition of Proliferation by PERK Regulates Mammary Acinar Morphogenesis and Tumor Formation

**DOI:** 10.1371/journal.pone.0000615

**Published:** 2007-07-18

**Authors:** Sharon J. Sequeira, Aparna C. Ranganathan, Alejandro P. Adam, Bibiana V. Iglesias, Eduardo F. Farias, Julio A. Aguirre-Ghiso

**Affiliations:** 1 Department of Biomedical Sciences, School of Public Health and Center for Excellence in Cancer Genomics, University at Albany, State University of New York, Rensselaer, New York, United States of America; 2 Division of Hematology/Oncology, Department of Medicine, Mount Sinai School of Medicine, New York, New York, United States of America; Ordway Research Institute, Inc., United States of America

## Abstract

Endoplasmic reticulum (ER) stress signaling can be mediated by the ER kinase PERK, which phosphorylates its substrate eIF2α. This in turn, results in translational repression and the activation of downstream programs that can limit cell growth through cell cycle arrest and/or apoptosis. These responses can also be initiated by perturbations in cell adhesion. Thus, we hypothesized that adhesion-dependent regulation of PERK signaling might determine cell fate. We tested this hypothesis in a model of mammary acini development, a morphogenetic process regulated in part by adhesion signaling. Here we report a novel role for PERK in limiting MCF10A mammary epithelial cell proliferation during acinar morphogenesis in 3D Matrigel culture as well as in preventing mammary tumor formation *in vivo*. We show that loss of adhesion to a suitable substratum induces PERK-dependent phosphorylation of eIF2α and selective upregulation of ATF4 and GADD153. Further, inhibition of endogenous PERK signaling during acinar morphogenesis, using two dominant-negative PERK mutants (PERK-ΔC or PERK-K618A), does not affect apoptosis but results instead in hyper-proliferative and enlarged lumen-filled acini, devoid of proper architecture. This phenotype correlated with an adhesion-dependent increase in translation initiation, Ki67 staining and upregulation of Laminin-5, ErbB1 and ErbB2 expression. More importantly, the MCF10A cells expressing PERKΔC, but not a vector control, were tumorigenic *in vivo* upon orthotopic implantation in denuded mouse mammary fat pads. Our results reveal that the PERK pathway is responsive to adhesion-regulated signals and that it is essential for proper acinar morphogenesis and in preventing mammary tumor formation. The possibility that deficiencies in PERK signaling could lead to hyperproliferation of the mammary epithelium and increase the likelihood of tumor formation, is of significance to the understanding of breast cancer.

## Introduction

Adhesion signaling is critical during mammary gland development where precise regulation of apoptosis and proliferation leads to proper tissue architecture and function [1]. For example, apoptosis of cells that detach from the basement membrane is required for the formation of the luminal space and overexpression of agonist-regulated dimerizing ErbB2 receptors disrupts this process and leads to multi-acinar structures devoid of a hollow lumen [2]. In addition, a distinguishing feature of breast cancers is the delocalized cell proliferation that leads to filling of the ductal lumen (i.e. DCIS) or complete loss of tissue architecture as observed in invasive carcinomas [3], [4]. Studies using a 3D *in vitro* MCF10A model of mammary acinar morphogenesis [4], as well as 2D adhesion vs. suspension growth assays revealed that loss of adhesion and lumen formation requires anoikis, a process that activates classical apoptotic mediators such as Bim [5]–[7]. However, other pathways may be activated to ensure proper lumen formation and their deregulation might lead to aberrant acinar development and subsequent tumor formation.

Early studies by Benecke et al., [8], [9] showed that fibroblasts that are denied attachment greatly repress translation initiation. This response can also lead to cellular quiescence [10], [11]. However, the mechanisms behind these responses were unknown. Translation initiation can be repressed by the 4EBP-dependent inhibition of the CAP-binding protein eIF4E or through the phosphorylation of the translation initiation factor eIF2α [12]. The latter is a target of kinases activated by different stimuli. For example, PKR or GCN2 can phosphorylate eIF2α in response to dsRNA or nutrient deprivation, respectively [13]. The endoplasmic reticulum (ER) kinase PERK can also phosphorylate eIF2α and repress translation initiation during stress conditions caused by unfolding of proteins [14]. PERK can induce growth arrest and/or apoptosis and has been linked to the induction of genes such as the transcription factor GADD153/CHOP [15], [16] or inhibition of cyclin D1 [17], [18]. Interestingly, ER stress signaling has been shown to be a negative regulator of malignancy in human squamous carcinoma cells [19], [20] and of H-Ras-mediated transformation of human melanocytes [21]. Further, inhibition of PKR and subsequent reduced phosphorylation of eIF2α was sufficient to cause transformation of mouse NIH3T3 fibroblasts [22]. These results suggest that phosphorylation of eIF2α could potentially have a tumor inhibitory function.

*In vitro* 3D Matrigel culture systems are useful for modeling the role of adhesion signaling during mammary acini lumen formation and filling [2], [23], [24] [3]. Interestingly, ATF4 and GADD153 (a target of ATF4) [25], two genes selectively upregulated by PERK signaling, are upregulated at different stages during mammary gland development [26], [27], suggesting that this pathway may be naturally regulated in this tissue. Further, loss of adhesion can strongly attenuate translation, a critical function of PERK [28], and signals that circumvent anoikis and stimulate proliferation can lead to lumen filling [29]. Thus, we hypothesized that adhesion-dependent regulation of PERK-eIF2α signaling for cell death and/or growth arrest may be important for acinar development and prevent aberrant growth. Given that PERK-eIF2α signaling can result in inhibition of proliferation or induction of apoptosis we explored these two possibilities as functional outputs of this pathway *in vitro* and *in vivo*.

## Results

It is known that loss of adhesion results in anoikis of epithelial cells [6] and this has been observed during *in vitro* acinar development [4]. Further, suspension growth assays have been very useful in elucidating the mechanistic intricacies linked to anoikis and acinar lumen formation in MCF10A cells [30]. Thus, we first used this standardized assay of adhesion vs. suspension growth, in order to gain insight into the link between the regulation of eIF2α phosphorylation and adhesion signaling.

### Adhesion Regulates The Phosphorylation of eIF2α and Protein Synthesis In MCF10A Cells

**Figure 1:**
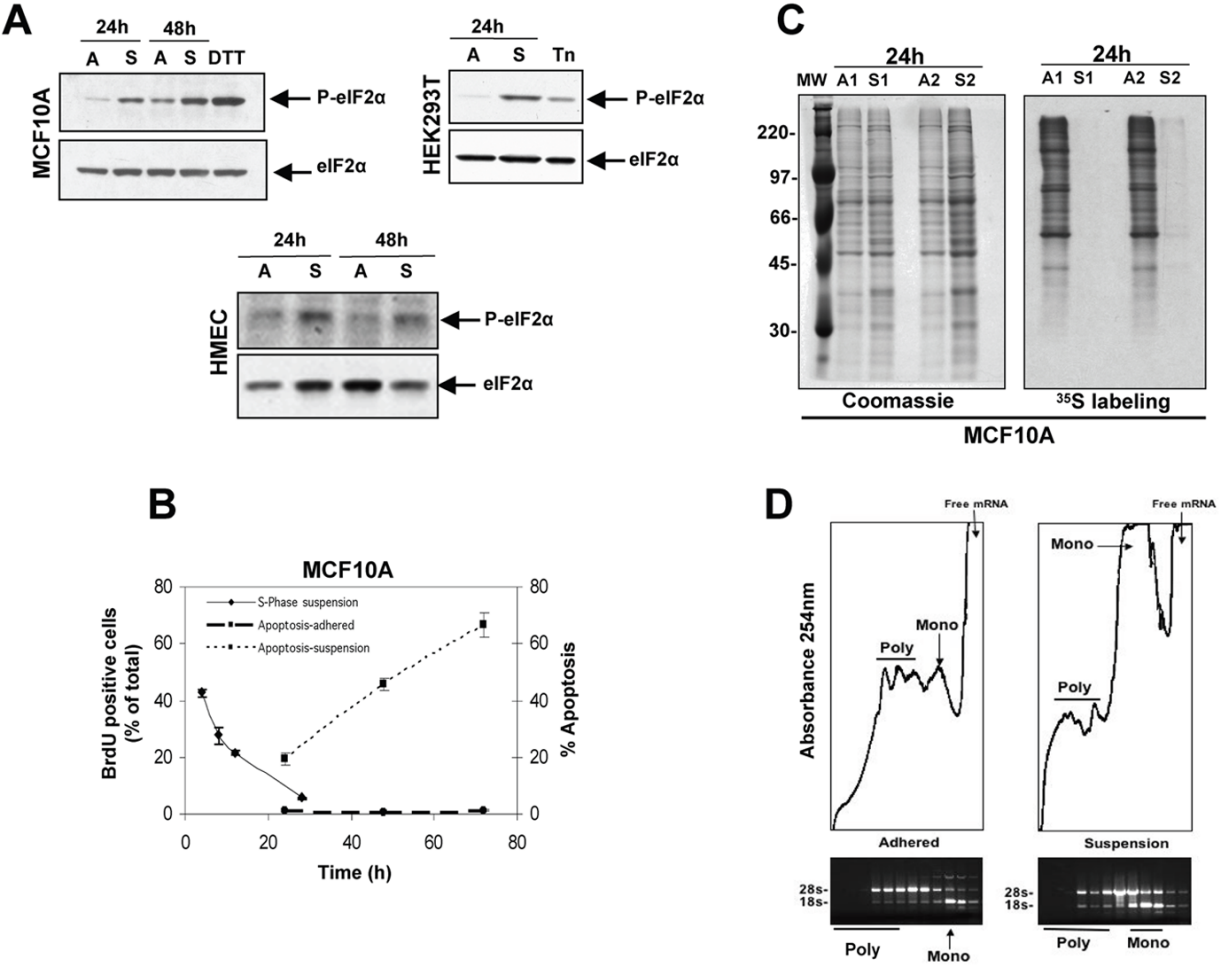
Suspension Induces Phosphorylation of eIF2α and Translation Repression in Mammary and Kidney Epithelial Cells. (A) Whole cell lysates from MCF10A (upper left), HEK293T (upper right) and primary HMEC (lower panels) cells grown either in adhered (A) or suspended conditions (S) as described in the methods section for the indicated time points, were immunoblotted for p-eIF2α and total eIF2α levels. Adhered MCF10A or HEK293T cells treated with 2 mM DTT or 5 µg/ml tunicamycin (Tn) respectively, were used as positive controls. (B) Quantification of the rate of DNA synthesis using a BrdU incorporation assay and flow cytometry to measure the percentage of BrdU-positive cells (filled diamonds) at different time points in suspension. The percentage of apoptotic cells was measured using propidium iodide staining and flow cytometry to identify the sub-G0 apoptotic fraction for adhered (dashed line) or suspended (dotted line) MCF10A cells for different time points. Data points show the mean±SD for BrdU–positive cells in each sample as a percentage of the total. (C) Autoradiogram of [^35^S] Met/Cys incorporation (right panel) into newly synthesized proteins in MCF10A cells adhered or suspended for 24 hrs (two independent samples). Coomassie Blue staining of an identical gel (left panel) shows equal protein loading. (D) Polysome profiles from 24 hr adhered (left) and suspended (right) MCF10A cells showing an increase and decrease in the monosome and polysome peaks, respectively in suspended cells. Absorbance at 254 nm (Y-axis, RNA concentration) was plotted against migration in the sucrose gradient (X-axis, bottom to top). Total RNA was isolated from individual fractions to visualize the 18S and 28S rRNAs by ethidium bromide staining.

We first determined whether the loss of adhesion might activate eIF2α phosphorylation at Ser51 (P-eIF2α), and if this response correlated with the growth arrest and apoptosis of MCF10A cells in suspension. MCF10A cells were detached by mild trypsinization or with PBS/2mM EDTA and after neutralization with media containing 5% horse serum, cells were either replated on tissue culture dishes (adhered conditions) or on dishes coated with agar in media containing 0.5% methylcellulose [30] for 24–48 hrs (suspension conditions) (**Fig 1A**). Western blots revealed that at 24 hrs in suspension there was increased phosphorylation of eIF2α at Ser51 (**Fig 1A**), which was detectable as early as 4 hrs in suspension (data not shown) and comparable to the signal induced by 2mM DTT treatment [31]. The time course of eIF2α phosphorylation in suspension correlated with the timing of withdrawal from S-phase and the subsequent onset of apoptosis in the same cells placed in suspension (**Fig 1B**). Primary human mammary epithelial cells (HMECs) and human kidney epithelial cells (HEK293T) also displayed increased levels of phosphorylated eIF2α when placed in suspension, suggesting that this is a conserved response in normal and immortalized epithelial cells (**Fig 1A**). We next tested whether phosphorylation of eIF2α correlated with inhibition of protein synthesis in MCF10A cells. Growth in suspension caused a robust attenuation of general protein synthesis as measured by ^35^S-Met incorporation (**Fig 1C**). This effect was comparable to the inhibition caused by 2mM DTT treatment and appeared as early as 4 hrs in suspension (data not shown). Similarly, relative to adhered cells, suspension conditions showed a significant decrease in the polysomes and a corresponding increase in the monosome peak (i.e. single 80s subunits) (**Fig 1D**). We conclude that suspension-induced eIF2α phosphorylation is associated with inhibition of translation initiation and this response correlates with an initial growth arrest followed by apoptosis of MCF10A cells.

### Induction of ATF4 and GADD153 in Suspension Is Not Accompanied by Chaperone Upregulation

**Figure 2:**
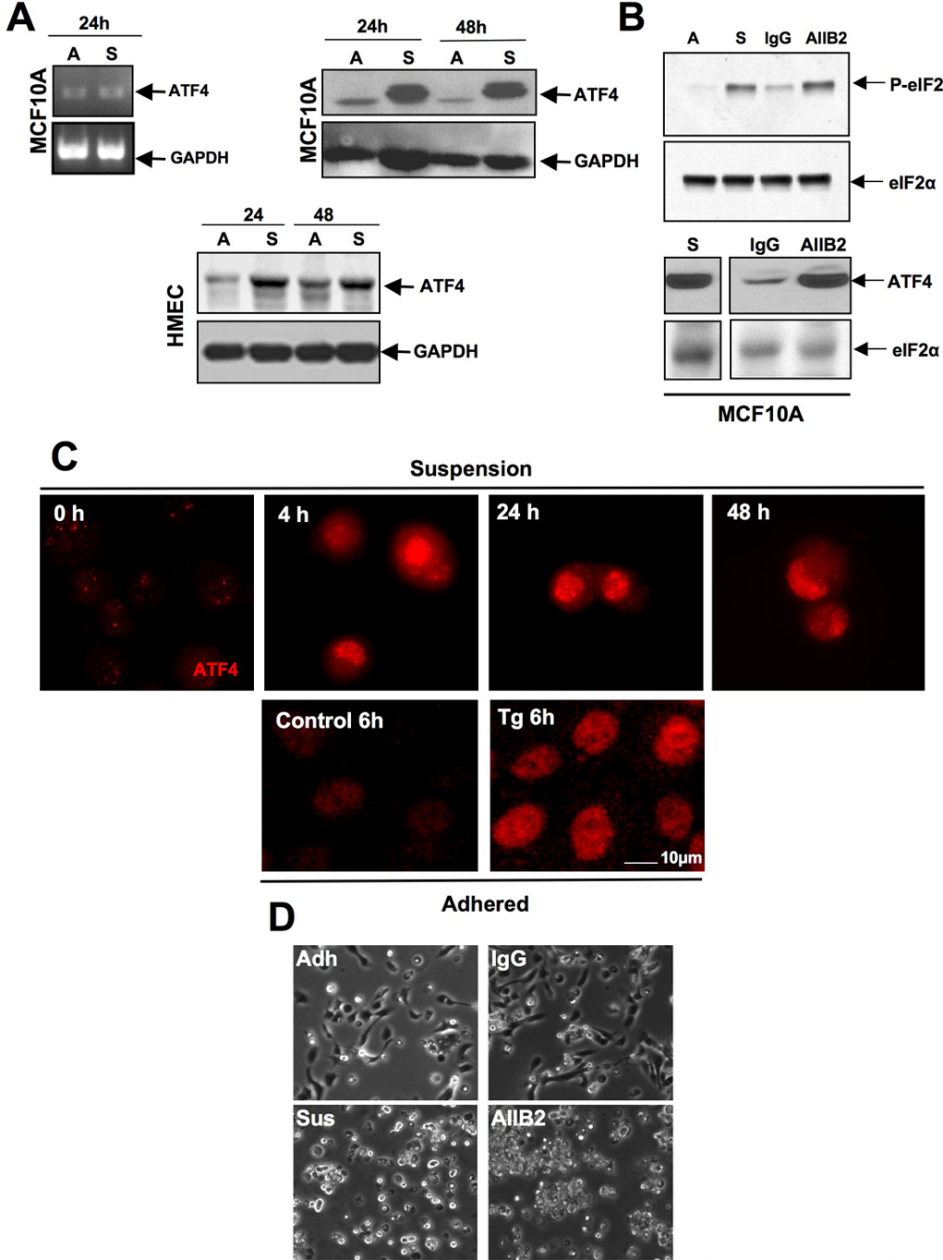
ATF4 Protein Levels Are Strongly Upregulated During Suspension Conditions. (A) RT-PCR analysis of ATF4 or GAPDH (as loading control) mRNA levels, in adhered (A) or suspended (S) MCF10A cells, (left panels); ATF4 protein levels or GAPDH (as loading control) in MCF10A (right panels) and primary HMEC cells (bottom panels) as detected by Western blotting. (B) Immunoblot showing increased levels of p-eIF2α (upper panels) or ATF4 (lower panels) protein comparable to that induced by suspension, in MCF10A cells pretreated with AIIB2, a β1-integrin function-blocking Ab (10 µg/ml). Control cells were treated with an isotype-matched IgG (10 µg/ml). Total eIF2α was used as loading control. (C) Immunofluorescent staining for ATF4 (red) in MCF10A cells immediately after detachment (0 hrs) and placed in suspension for the indicated time points or treated with thapsigargin (4 µM) for 6 hrs before fixing on poly-lysine-coated coverslips to facilitate detection. (D) Photomicrographs of MCF10A cells plated on Laminin-1 coated tissue culture dishes, pre-incubated with AIIB2 (10 µg/ml) or a control IgG antibody (10 µg/ml) prior to plating or placed in suspension on agar-coated dishes in the presence of 0.5% methylcellulose in media containing 1% serum.

Concomitant to general translation repression, phosphorylation of eIF2α results in preferential translation of the ER stress regulated transcription factor ATF4 while the mRNA levels remain constant [25]. We therefore determined whether accumulation of ATF4 protein occurred in the absence of an increase in mRNA levels in adhered vs. suspension conditions. MCF10A cells grown adhered or in suspension for 24 hrs showed no increase in the levels of ATF4 mRNA (**Fig 2A**). In contrast, detection of ATF4 protein by Western blot showed a strong increase in ATF4 protein levels at 24 and 48 hrs (**Fig 2A**) in suspended MCF10A and HMEC cells, suggesting a translational enhancement of ATF4 message. This was confirmed with experiments showing that the ATF4 mRNA was preferentially enriched in heavy polysome fractions only in MCF10A cells kept in suspension (data not shown). We further tested whether cells in suspension showed ATF4 protein accumulation as measured by IF. These results (**Fig 2C**) show that as early as 4 hrs and as late as 24 and 48 hrs post-suspension, ATF4 protein expression increased and displayed a nuclear localization pattern. Thapsigargin treatment used as a positive control also increased ATF4 expression and nuclear localization. An anti-HA rabbit polyclonal Ab used as a negative control showed no staining (data not shown). Together these results strongly suggest that loss of anchorage of normally adherent cells initiates stress signals that result in eIF2α phosphorylation and subsequent upregulation of ATF4 protein expression.

We next determined whether integrins were required to regulate eIF2α phosphorylation. To this end we used a β1-integrin function-blocking monoclonal antibody (AIIB2) previously shown to disrupt cell adhesion [24]. MCF10A cells were pre-incubated in suspension with 10 µg/ml AIIB2 antibody (**Fig 2B**) for 30 min at 37°C and then allowed to adhere to various matrices including 10 µg/ml Laminin-1 (LN-1). We found that while a control IgG had no effect on adhesion to LN-1 (**Fig 2D**) or Matrigel (data not shown), treatment with the AIIB2 antibody resulted in almost complete cell rounding and detachment accompanied by an increase in phosphorylation of eIF2α and upregulation of ATF4 (**Fig 2B and 2D**). These results further strengthen our hypothesis that adhesion signals mediated by ligand-bound integrins are required to maintain eIF2α phosphorylation at basal levels.

**Figure 3:**
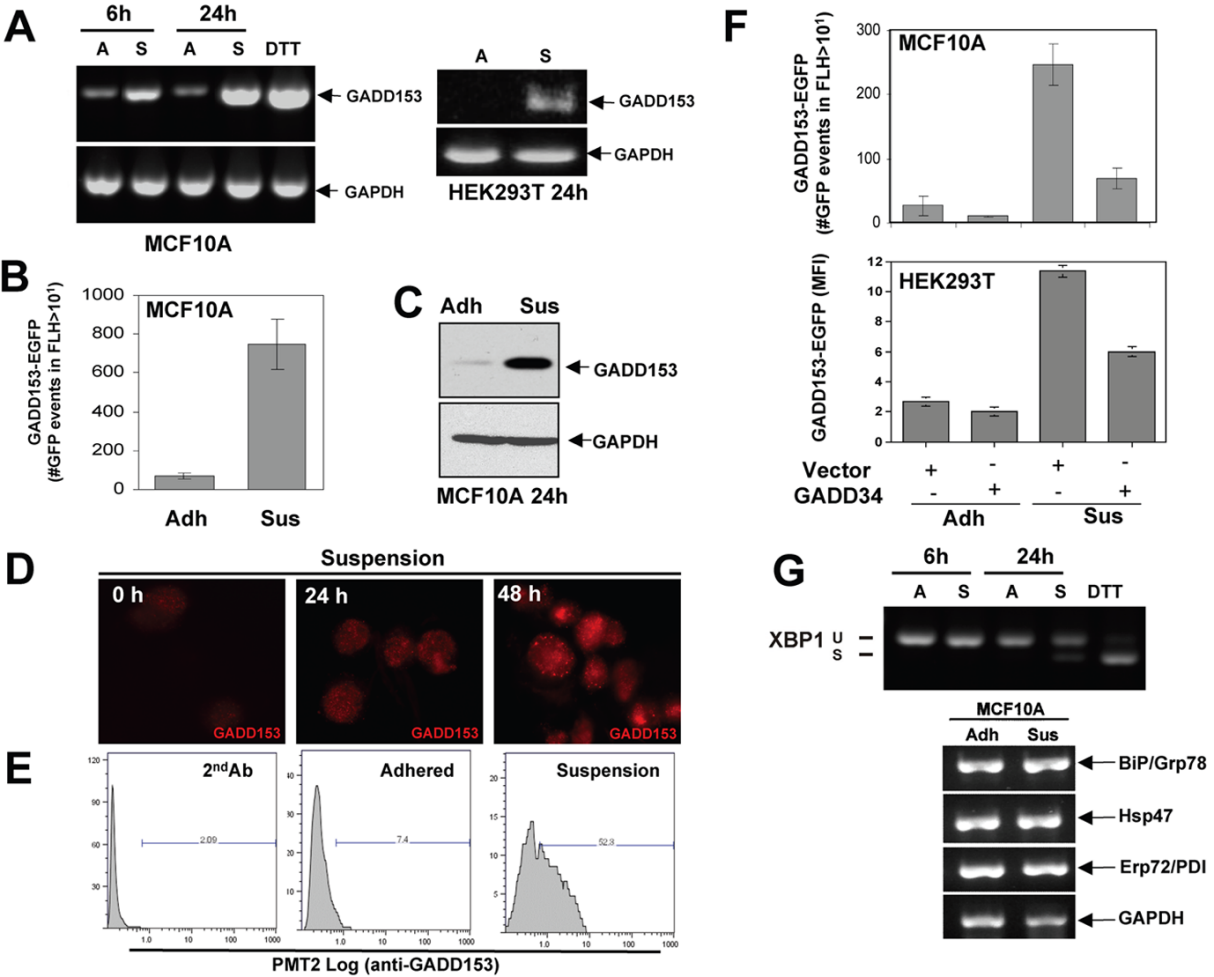
GADD153 mRNA and Protein Levels Are Strongly Upregulated During Suspension Conditions. (A) RT-PCR analysis of GADD153 mRNA levels in MCF10A (left panel) and HEK293T (right panel) cells at different time points in either adhered (A) or suspended (S) conditions. Adhered MCF10A cells treated with 2mM DTT for 4h were used as positive control and GAPDH was used as a loading control. (B) MCF10A cells were transiently transfected with a GADD153 promoter-driven-EGFP reporter plasmid and EGFP fluorescence was analyzed 48 h post-transfection by FACS; total events captured: 2×10^4^. The graph shows the number of GFP-positive events in FLH2>10 (mean±SD). (C) Western blot for GADD153 protein in adhered (A) or suspended (S) MCF10A cells. (D and E) Immunofluorescence (D) and FACS (E) analysis of GADD153 (red) expression in MCF10A cells following growth in adhered or suspension conditions for the indicated times. Secondary antibody was used as negative control in E. (F) MCF10A (top) and HEK293T (lower) cells were transiently co-transfected with the GADD153-EGFP reporter plasmid and either a full-length Flag-tagged GADD34 plasmid or an empty vector as control for 24 hrs before being detached and left to reattach or put into suspension for an additional 48 hrs before FACS analysis. GFP fluorescence was analyzed 48 h post transfection by FACS where a total 2×10^4^ events were captured. The graphs show the number of GFP positive events in FLH2>10 or the mean fluorescence intensity (MFI) in the PMT2-FITC channel (mean±SD). (G) RT-PCR for XBP-1 splicing (top panel) in MCF10A cells at different time points either adhered (A) or suspended (S). Adhered MCF10A cells treated with 2mM DTT for 4 hrs was used as positive control and GAPDH, shown in (A) was used as a loading control. Lower panels show RT-PCR for BiP, Hsp47 and Erp72/PDI chaperone mRNA levels in adhered (Adh) or suspended (Sus) MCF10A cells. GAPDH was used as a loading control.

We next determined whether the growth-arrest and DNA-damage 153 (GADD153) gene [15], [32] [33], an ATF4 target, was upregulated in suspension [34]. MCF10A cells placed in suspension showed increases in GADD153 mRNA as early as 6 hrs and a strong upregulation of the mRNA and protein were also detected at 24 hrs (**Fig 3A and C**). IF studies revealed a similar increase that was quantitated by FACS (**Fig 3D and E**). We also detected a 6-fold upregulation of GADD153 promoter activation measured using a GADD153 promoter-driven EGFP reporter construct and flow cytometry (**Fig 3B**). In HEK293T cells both GADD153-EGFP reporter activity and endogenous GADD153 mRNA were increased after cells were placed in suspension for 24 hrs (**Fig 3A and3F**). Interestingly, in both MCF10A and HEK293T cells, GADD153-EGFP reporter activity was downregulated by overexpression of GADD34, a regulatory subunit of the phosphatase PP1C that dephosphorylates eIF2α [35], suggesting a dependence on eIF2α phosphorylation (**Fig 3F**). We also detected a weak increase in XBP-1 splicing [36] in suspension (**Fig 3G** **upper panel**), which was followed by a marginal increase in protein levels (data not shown). However, in contrast to ATF4 and GADD153, this increase was only detectable at 24 hrs and not comparable to that induced by 2mM DTT treatment. This suggests that XBP-1 splicing may not be a major pathway activated by suspension growth conditions or that it may be activated later, when the vast majority of the cells are committed to apoptosis. Unlike GADD153 expression, no significant changes were observed in suspension for the expression of canonical ER stress-induced response genes such as the chaperones, BiP/Grp78, Hsp47 or PDI/Erp72 (**Fig 3G** **, lower panels**) [37]. The lack of upregulation of these markers suggests that while suspension growth assays induce eIF2α phosphorylation, ATF4 and GADD153 expression and to a lesser extent XBP-1 splicing, these changes do not immediately impact the expression of some UPR-regulated chaperones.

### Suspension-Induced Phosphorylation of eIF2α at Ser-51 Involves PERK Activation

**Figure 4:**
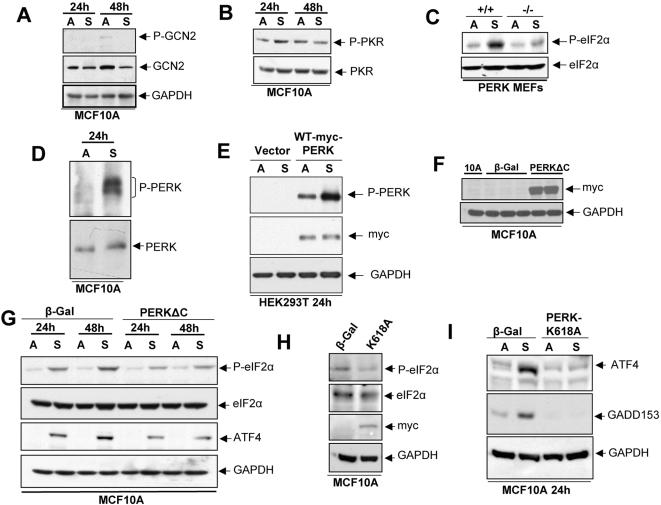
PERK is the Major Upstream Kinase Mediating Phosphorylation of eIF2α in Suspension. (A and B) Immunoblot for phospho- and total GCN2 and PKR in MCF10A cells in adhered or suspension conditions for 24 and 48 hrs. Note that only a faint phospho-protein band for phospho-GCN2 was observed at the 220 kD position but in adhered conditions. GAPDH was used to show equal loading. (C) IB for phospho-(top blot) and total- (bottom blot) eIF2α in PERK wild-type and PERK-/-MEFs grown in adhesion (A) or in suspension (S) for 24 hrs. (D) IB for phospho- and total PERK in MCF10A cells grown adhered (A) or in suspension (S) for 24 hrs. (E) IB for phospho- PERK and myc in HEK293T cells transiently transfected with empty vector control or mouse WT-myc tagged-PERK and placed in adhered (A) or suspended (S) conditions for 24 hrs. GAPDH serves as loading control. (F) IB for Myc-PERKΔC expression in parental and in MCF10A cells stably expressing either pBabepuro-myc-PERKΔC or a vector control pBabepuro-β-galactosidase construct. (G) IB for phospho- and total eIF2α and ATF4 expression in β-Gal or myc-PERKΔC expressing cells either adhered or suspended for 24 and 48 hrs. GAPDH was used as loading control. (H) IB for Myc, phospho and total eIF2α and GAPDH in MCF10A cells stably expressing β-Gal (vector control) or a pBabeneo-PERK-K618A (kinase dead) mutant. (I) Cells obtained in (H) were used in a suspension vs. adhesion assay for 24 hrs and ATF4 and GADD153 expression was detected through IB in the cell lysates. GAPDH was used as a loading control.

The above results were intriguing because they suggested that changes in adhesion might regulate ER kinases or other signaling pathways that result in phosphorylation of eIF2α. Thus, we next determined whether GCN2, PKR or PERK were kinases potentially responsible for the increase in eIF2α phosphorylation in suspension. While total GCN2 protein levels were abundant and slightly higher in adhered vs. suspension conditions at 48 hrs (GAPDH shows equal loading), we found very low levels of phospho-GCN2 that were not modulated by suspension at these time points (**Fig 4A**). GAPDH was used as an additional loading control. Similarly, suspension growth conditions did not significantly affect phospho- or total-PKR levels (**Fig 4B**). The expression of another eIF2α kinase, the heme-regulated initiation factor 2α kinase (HRI) is mostly restricted to cells of the hematopoietic lineage [38]. Thus, we focused on PERK as a potential transducer of suspension-induced eIF2α phosphorylation.

To test the link between adhesion and PERK signaling we determined whether *WT* or *PERK*-/-MEFs [14] displayed differential phosphorylation of eIF2α in suspension. As shown in **Fig 4C**, *WT*-MEFs placed in suspension for 24 hrs showed a robust phosphorylation of eIF2α at Ser51. Strikingly, this response was greatly reduced in *PERK*-/-MEFs, suggesting that PERK is an important contributor to eIF2α phosphorylation in response to cell detachment in mouse cells (**Fig 4C**). We next tested PERK phosphorylation in MCF10A cells. Our results in **Fig 4D** show that after 24 hrs in suspension there is a strong phosphorylation of PERK, while almost no signal can be detected in adhered conditions for the same time. This correlates with the robust phosphorylation of eIF2α shown in **Fig 1A** **and** **2B**. Total PERK levels appeared unchanged in adhered vs. suspension conditions. A slightly slower mobility of PERK protein in suspension is probably indicative of the phosphorylation. Of note is the fact that our attempts to reliably detect any endogenous phospho-PERK levels in HEK293T cells by Western blotting were unsuccessful even in the presence of ER stressors such as DTT, tunicamycin or thapsigargin. Thus, to further support that PERK activation can be detected in these epithelial cells in suspension and that this correlates with the increase in P-eIF2α, we relied on the detection of overexpressed PERK. To this end, HEK293T cells, which display eIF2α phosphorylation in suspension (**Fig 1A**) were transfected (∼100% transfection efficiency) with a mouse m*yc*-tagged *wtPERK* for 48 hrs and then placed in adhered vs. suspension conditions for 24 hrs. As mentioned above, vector transfected cells showed no detectable P-PERK signals (**Fig 4E**). In contrast, adhered HEK293T cells expressing wtPERK displayed detectable PERK phosphorylation that, unlike GCN2 and PKR, was strongly increased in suspension (**Fig 4E**). To functionally test the role of PERK signaling in MCF10A cells we used genetic approaches to target this pathway. We retrovirally transduced these cells to stably express a pBabe-puro construct encoding a Myc-tagged dominant negative PERK where the C-terminal cytoplasmic kinase domain is deleted (PERKΔC) (**Fig 4F–G**) or a mutant where Lys-618 was mutated to Ala, which results in a kinase dead (PERK-K618A) enzyme (**Fig 4H–I**). Control MCF10A cells were transduced with a virus encoding pBabe-puro plasmid encoding β-galactosidase as vector control. In all experiments P-eIF2α, ATF4 (**Fig 4G,H,I**) and GADD153 (**Fig 4I**) were used as a read-out of PERK activity. Expression of the dominant negative PERKΔC resulted in inhibition of the increase in P-eIF2α, ATF4 in suspension (**Fig 4G**) without significantly affecting total eIF2α levels. GAPDH was used as a loading control. Similarly expression of the kinase dead PERK-K618A mutant also inhibited basal eIF2α phosphorylation (**Fig 4H**). In addition, suspension-induced ATF4 and GADD153 was inhibited in MCF10A cells expressing PERK-K618A (**Fig 4I**). Together, our results demonstrate that the signal that results in eIF2α phosphorylation and upregulation of downstream targets by loss of adhesion of MCF10A cells is largely dependent on PERK.

### PERK Inhibition Deregulates Mammary Acinar Development in 3D Matrigel

Our experiments using 2D culture revealed a strong functional link between adhesion and PERK signaling. We next determined the functional output of PERK signaling in MCF10A cells using 3D Matrigel cultures, an assay that recapitulates the physiological context offered by normal tissue architecture and a process in which adhesion signaling is critical for proper acinar development [2], [23], [24], [39]. MCF10A cells cultured in 3D Matrigel proliferate and go on to form well organized polarized acini and cells that detach from the basement membrane undergo anoikis favoring lumen formation [39]. In addition, approximately by day 10 of development these structures reach their size limit [4]. 3D cultured MCF10A cells were analyzed through immunofluorescence (IF) and standard or laser scanning confocal microscopy (LSCM). Control experiments revealed that as reported [4], these cells formed at day 12 hollow acini that displayed basal deposition of laminin-5 (LN-5) around the colony, GM130 (a Golgi marker protein) localized mostly to the luminal region of the acini where cleaved (active) caspase-3 positive cells were also observed (data not shown).

**Figure 5:**
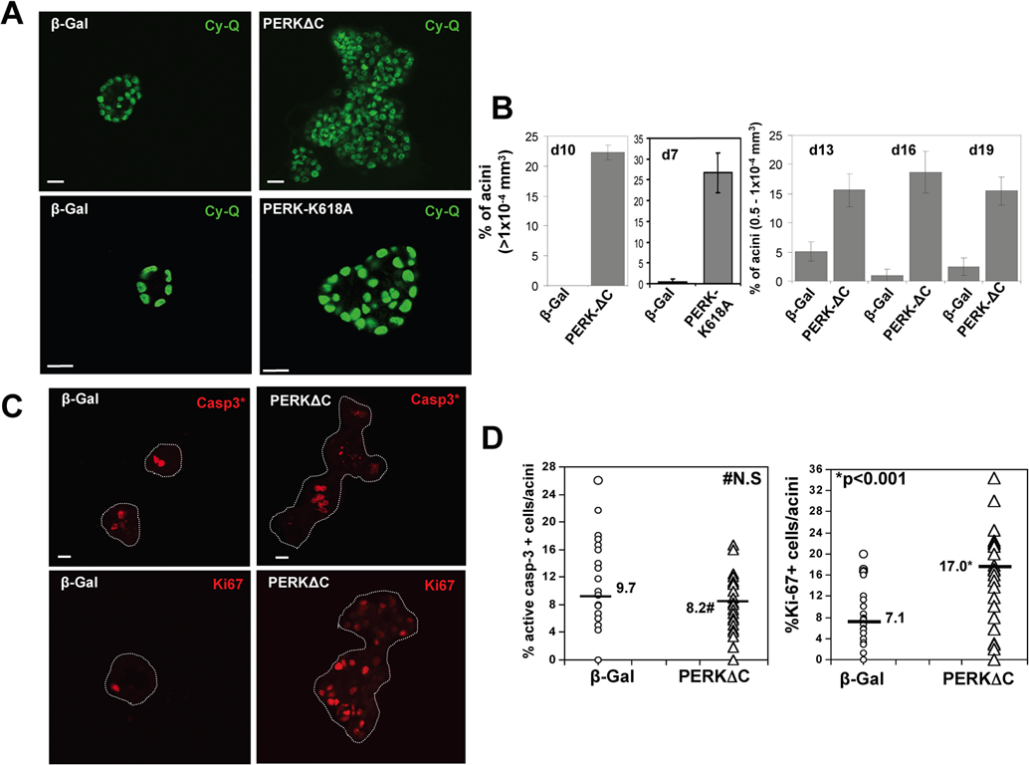
PERK is Required to Maintain Normal Mammary Acini Growth and Morphogenesis through Regulation of Cell Proliferation. (A) Representative images through the equatorial cross sections of acini from myc-PERKΔC, myc-PERK-K618A or β-gal expressing MCF10A cells on Day 8 of culture in 3D Matrigel, using LSCM. Nuclei are stained green using CyQuant dye. Note the increase in acinar size, luminal filling and the multi-lobular nature of the PERKΔC structures as compared to the vector control acini. PERK-K618A expressing acini were also significantly larger and always showed luminal cells, but were not frequently multi-lobulated. Scale bars = 20 µm. (B) Acinar size was measured on different days in Matrigel for β-Gal and PERKΔC or PERK-K618A cells, using SPOT™ software to measure two perpendicular diameters per acinus and calculate acinar volume considering an ellipsoid structure; volume for 50–200 acini were quantitated per time point, shown as percentage of total for the given size range, Mean±SD. (C) Confocal images for β-Gal and PERKΔC cells immunostained for cleaved (*) caspase-3 (red, top panels) or Ki-67 (red, bottom panels) on Day 8 in Matrigel. Acini are outlined with white dotted lines to delineate the normal β-Gal vs. disorganized large PERKΔC acini, which shows Ki-67 positively stained cells in the lumen. Scale bars = 20 µm. (D) Graphs showing distribution of the percentage of cells per acinus that stained positively for Ki-67 or cleaved caspase-3 in β-Gal and PERKΔC cells on Day 8 in Matrigel; between 50–100 acini were scored and statistical significance was determined by the *t* test for independent samples with P<0.001 defined as statistically significant. #N.S-not significant.

**Figure 6:**
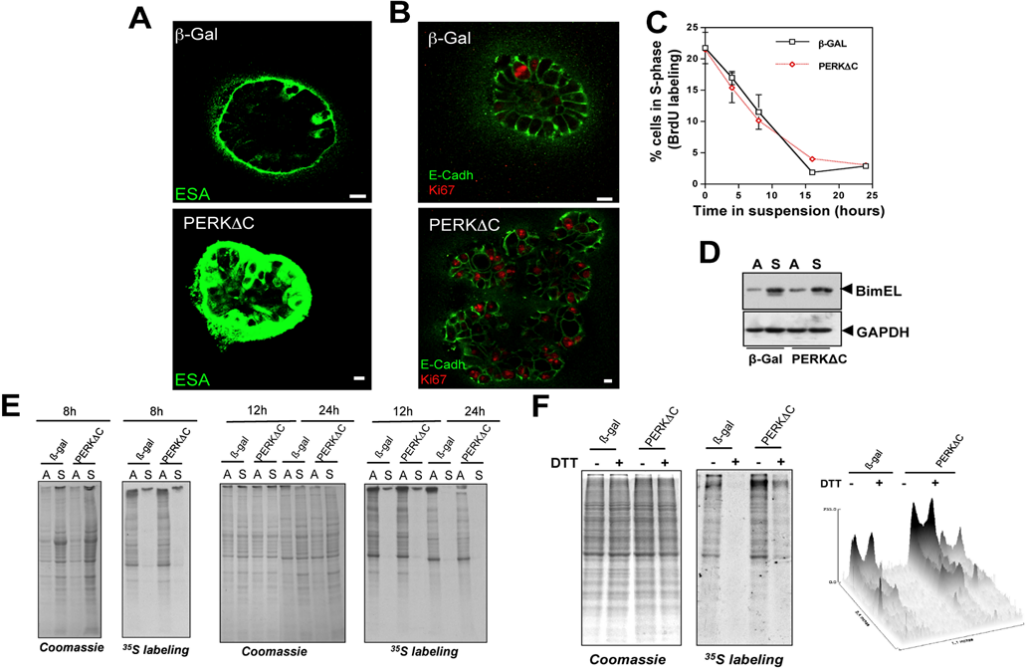
Immunofluorescence for ESA and E-cadherin In β-Gal Control and PERKΔC Acini. (A) Representative confocal images of cross sections through β-Gal and PERKΔC acini immunostained for the cell surface glycoprotein, ESA (epithelial specific antigen, green), on Day 8 in Matrigel. Note the presence of ESA in cells occupying the luminal space of PERKΔC acini (bottom) as compared to proper basolateral localization of ESA in the β-Gal control acini (top). (B) Representative confocal images of β-Gal and PERKΔC acini co-stained for E-cadherin (green) and Ki-67 (red), on Day 8 in Matrigel. Note the presence of strong E-cadherin staining in the cell-cell junctions of luminal cells in PERKΔC acini that also stain positive for Ki-67 while β-Gal control acini show little to no Ki-67 staining in luminal cells and the expected apicolateral staining for E-cadherin in the outer rim of cells. Scale bars = 10 µm. (C) Proliferation assay using BrdU incorporation and flow cytometry to measure the percentage of BrdU-positive β-Gal or PERKΔC cells at different time points in suspension, mean±SD. (D) Immunoblot showing an increase in BimEL levels in suspension but no significant change between β-Gal and PERKΔC cells, GAPDH serves as loading control. (E) Autoradiogram of [^35^S] Met/Cys incorporation into newly synthesized proteins in β-Gal or PERKΔC cells in full (5%) serum media placed in adhered or suspended growth conditions for 8 hrs, 12 hrs or 24 hrs, showing complete attenuation of protein synthesis in suspension. Coomassie Blue staining of identical gels (left panels) shows protein loading. (F) Autoradiogram of [^35^S] Met/Cys incorporation into newly synthesized proteins in adhered β-Gal or PERKΔC cells placed in low (1%) serum media, treated with or without DTT (2 mM for 1hr), showing increased protein synthesis in PERKΔC cells. Coomassie Blue staining of an identical gel (left panel) shows equal protein loading. Densitometric analysis and surface plotting of the bands illustrates the increase in intensity in the PERKΔC cells.

MCF10A cells stably expressing β-Gal, PERKΔC or PERK-K618A dominant negative mutants were cultured in 3D Matrigel as described above. Strikingly, as early as 8 days in 3D culture and throughout the assay until day 19, we observed that PERKΔC expressing cells formed significantly larger acini that had an amorphous multi-lobular architecture (**Fig 5A**). Staining for epithelial specific antigen (ESA) [40] or E-cadherin showed the expected baso- or apico-lateral distribution in β-Gal cells, respectively (**Fig 6A–B**). In contrast, PERKΔC cells appeared to have lost epithelial organization as the staining for ESA, in addition to being stronger appeared disorganized and was present in the outer rim of cells and also in luminal cells (**Fig 6A**). E-cadherin was also distributed in an apico-lateral pattern in β-Gal cells, while in PERKΔC cells a disorganized distribution was observed, which appeared uniform around the cells and sometimes basal, suggesting an alteration in polarity (**Fig 6B**). A similar phenotype to that resulting from PERKΔC expression was observed for PERK-K618A expressing cells that showed larger acini with irregular architecture (**Fig 5A**). We next analyzed the size differences between β-Gal, PERKΔC and PERK-K618A acini by measuring two perpendicular diameters and calculating the volume of individual acini considering an ellipsoid morphology. We observed that the frequency of PERK-mutant acini in and above the 0.5–1×10^−4^ mm^3^ volume ranges was 5-10-fold higher than the β-Gal control cells (**Fig 5B**). Our results reveal that inhibition of PERK during mammary acini development in 3D results in a deregulation of the process leading to larger multi-acinar structures devoid of normal architecture and with a filled lumen.

### PERK Inhibits Proliferation During Acinar Morphogenesis

The increase in PERKΔC acini size could result from increased cell division, reduced apoptosis or a combination of both. To test whether the inhibition of PERK had any effect on the rate of apoptosis, we stained the acini on day 8 and 19 for cleaved caspase-3. Our results revealed that on day 8 relative to the β-Gal control cells PERKΔC expressing cells displayed only a marginal trend towards reduced apoptosis (**Fig 5C, top row and 5D**). Similar results were observed at day 19 (data not shown). These results suggest that PERK is not required for the activation of caspase-3 and apoptosis during MCF10A acini formation.

We next stained for Ki67 in the acini, as uncontrolled proliferation not counterbalanced by increased apoptosis could account for the larger number of cells and acini size. Through IF, we scored the number of cells per acini that were positive for Ki67 in β-Gal and PERKΔC cells on day 8 in Matrigel. These data revealed that inhibition of PERK signaling through the PERKΔC construct resulted in an ∼2.4 fold increase in Ki67 positive cells per acinus (*p*<0.001) (**Fig 5C bottom row and 5D**), than the β-Gal control acini. Ki67 positive cells in PERKΔC acini were located both in the rim of the colonies and prominently in the cell-filled lumen (**Fig 5C, bottom row and 5D**). These results allow us to conclude that the importance of PERK signaling during acini formation is in limiting cell proliferation, rather than apoptosis.

Based on the finding that PERK inhibition does not affect caspase-3 activation in luminal cells which undergo anoikis, we predicted that PERK signaling would not affect 2D suspension-induced apoptosis. In addition, if translational repression is important for anoikis, then PERK inhibition should not affect this process. To test this prediction we determined whether PERK signaling was linked to suspension induced cell cycle exit, apoptosis and translational repression. As predicted by our 3D morphogenesis assays, suspension-induced S-phase withdrawal and subsequent apoptosis were not affected by the expression of PERKΔC (**Fig 6C** **and data not shown**). BimEL is a proapoptotic protein required for acinar lumen formation [41]. Our data showed equal levels of BimEL upregulation in both β-Gal and PERKΔC cells in suspension (**Fig 6D**), which appears to be insensitive to the attenuation of translation or is highly stable. Furthermore, ^35^S-Met incorporation assays comparing adhered vs. suspended β-Gal and PERKΔC cells in full serum (5%) revealed that PERK inhibition under these conditions does not alleviate translational suppression (**Fig 6E**). These results suggest that suspension-induced apoptosis is not dependent on PERK and this is in agreement with the caspase-3 activation during acinar morphogenesis.

As shown in **Fig 5** and reported previously, proliferation is mostly restricted to the basal compartment of the normal acini. Thus, it could be assumed that in contrast to β-Gal acini, which do not increase in size beyond a certain point (60–70 µm after day 10), cells expressing PERKΔC may be unable to repress translation, favoring proliferation in 3D cultures and subsequent expansion of these structures. To test this prediction we determined whether translation initiation was affected in adherent β-Gal or PERKΔC cells. The 3D assays were performed in low serum (2% v/v). Thus, we tested whether adhered PERKΔC cells placed in low serum (1% v/v), which has been shown to result in reduced translation initiation [42], might be capable of enhanced ^35^S-Met incorporation. Adhered PERKΔC cells grown in these conditions had a higher basal ^35^S-Met incorporation than β-Gal cells. Further, in contrast to control cells, PERKΔC cells were partially resistant to DTT-mediated inhibition of translation (**Fig 6F**). Together, our 2D and 3D assay results suggest that under normal growth conditions PERK functions to inhibit translation initiation in adhered conditions and that this is associated with reduced proliferation, but not increased apoptosis, during acini development.

**Figure 7:**
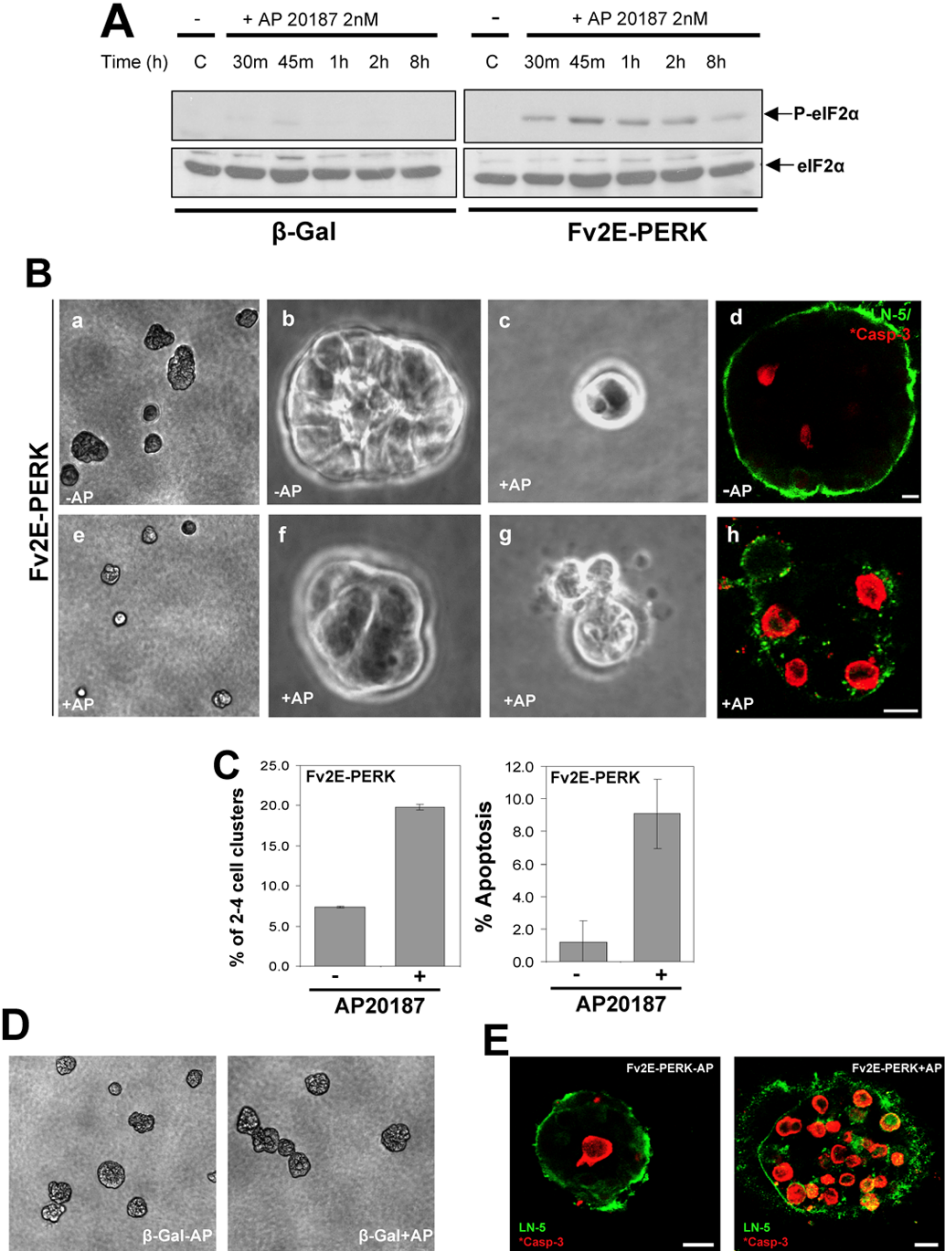
Unscheduled Activation of PERK Restricts Acinar Growth and Promotes Apoptosis in 3D Matrigel. (A) Time-dependent increase in phosphorylation of eIF2α in MCF10A cells expressing an Fv2E-ΔNPERK construct, upon treatment with the dimerizing drug, AP20187 (2 nM). AP20187 has no effect on P-eIF2α levels in β-Gal cells. Total eIF2α was used as loading control. (B) Photomicrographs of Fv2E-ΔNPERK cells in 3D Matrigel treated with 2 nM AP20187 or equal volume of ethanol as control, added every 24 hrs from Day 4 up to Day 6 of morphogenesis; representative phase-contrast images depict the effect of forced PERK activation on acini development; (B-a and B-e) A×10 magnification image of several developing acini; (B-b) Normal acinus, (B-c) 2 cell cluster, (B-f) 4 cell cluster, (B-g) 4 cell cluster containing apoptotic cells. (B-d and h) Confocal images through the equatorial region of Fv2E-ΔNPERK cells in 3D Matrigel immunostained for active caspase-3 (red) or LN-5 (green) with (B-h) and without (B-d) treatment with 2nM AP20187 every 24 hrs, (B-h) cell cluster where the majority of cells have entered apoptosis. (C) Quantitation of phase contrast images of Fv2E-ΔNPERK cells on Day 6, treated every 24 hrs with or without 2nM AP20187. Over 400 acini were visually scored for the presence of apoptotic or growth arrested 2–4 celled acini and calculated as a percentage of the total number of acini; graph shows mean±SD. (D) Photomicrographs of β-Gal vector control cells treated with 2nM AP20187 or with equal volume EtOH as control, every 24 hrs from Day 4 up to Day 6 in Matrigel. Note that AP20187 treatment caused no noticeable changes in acini size or morphology, consistent with the absence of modulation of P-eIF2α levels in the same cells (*A*). (E) Confocal images showing Fv2E-ΔNPERK cells treated with (+AP) or without (-AP) AP20187 every 12 hrs stained for LN-5 (green) to delineate the acini and active caspase-3 (red). Note that a majority of cells even in large acini can be pushed into apoptosis by strong activation of PERK signaling. Scale bars = 10 µm.

Further support that activation of PERK can strongly inhibit acini formation was obtained by activating PERK signaling in MCF10A cells stably expressing an Fv2E-ΔNPERK construct (Fv2E-PERK) [43]. In these cells the Fv2E dimerization domain is fused to the cytoplasmic kinase domain of PERK [43] and the kinase activity and translational repression is induced by forcing dimerization using the divalent compound AP20187, in the absence of ER stress [43]. Upon treatment with AP20187 (2nM), adhered Fv2E-PERK expressing cells showed a sustained increase in eIF2α phosphorylation between 0.5–1 hrs and the signal was lower but still sustained by AP20187 treatment at 8 hrs (**Fig 7A**). This response was not detected in β-Gal control cells (**Fig 7A**). β-Gal and Fv2E-PERK expressing cells cultured in 3D Matrigel were allowed to develop and at day 4, 2nM AP20187 was added every 12 or 24 hrs. The results showed that two days later, AP20187 treatment every 24 hrs had no influence on β-Gal acinar morphogenesis (**Fig 7D**), but inhibited acini development of Fv2E-PERK cells (**Fig 7B–C**). While only 7% of the vehicle (EtOH) treated cells were at the 2-4-cell stage on day 6 (**Fig 7C**), approximately 20% of the Fv2E-PERK cells treated with AP20187 were still at this stage (**Fig 7C**). However, except for ∼5% of the FV2E-PERK cells that remained as single cells regardless of the treatment, the rest of the AP20187 treated cells (75%) were able to escape the treatment and progress into larger (>8 cells) acini. We also observed at day 6, when apoptosis is not fully initiated, that Fv2E-PERK acini treated with AP20187 every 24 hrs showed a 10 fold increase in apoptotic cells, as compared to controls (**Fig 7B–C**). Apoptosis was further enhanced by treating the acini every 12 hrs as detected by increased active caspase-3 staining in the majority of cells in the acini (**Fig 7E**).Apoptosis may result from the strong activation of Fv2E-PERK caused by 2 nM AP20187 as lower doses induce growth arrest without apoptosis (data not shown). Together our results strongly support the notion that PERK is required to limit acini development and that positive or negative perturbations in PERK signaling can lead to either blockade or hyper-proliferative acinar morphogenesis, respectively.

### PERKΔC acini display reduced GADD153 expression and deregulated Laminin-5 production and organization

We next tested whether the expression of GADD153 [15], [32] and the levels of laminin-5, a component of the BM of the mammary acinus [44] were affected by PERK inhibition.

**Figure 8:**
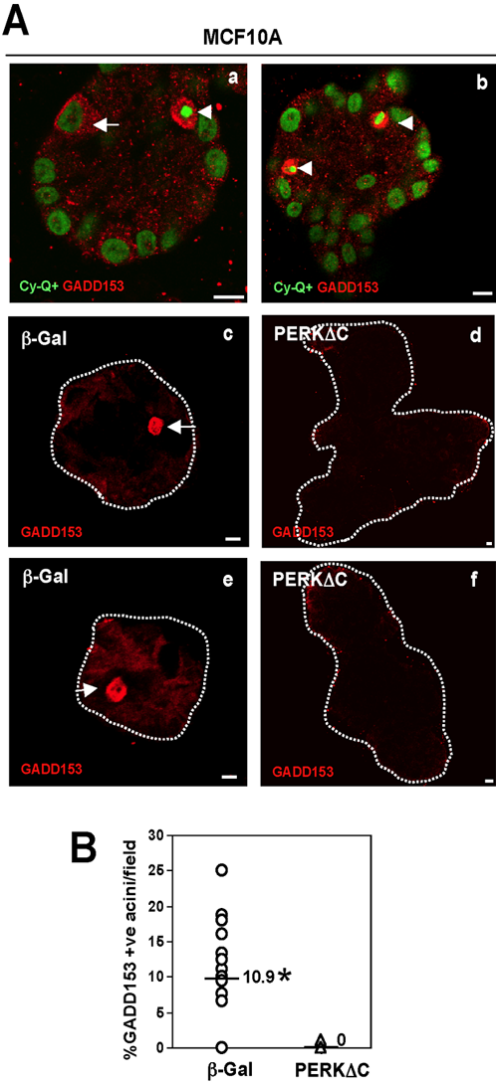
GADD153 Upregulation During Acini Development in 3D Cultures is PERK-dependent. (Aa and Ab) Representative confocal images through the equatorial section of Day10 MCF10A acini depicts strong GADD153-positive staining (red) in cells in the luminal space (arrowheads) that also display condensed nuclei, co-stained with CyQuant (green) as well as in some cases, in cells that localize to the outer basal layer of cells (arrow) that do not have condensed nuclei. (Ac-f) Representative images of GADD153 staining (red) in β-Gal (Ac and Ae) and PERKΔC (Ad and Af), cells on Day 8 in Matrigel. Scale bars = 10 µm. (B) Graph showing the distribution and median of GADD153- positive acini/field; 21 fields adding up to a total of 215 acini were scored.* P<0.001 determined by *t* test for independent samples.

We found that while few cells in control β-Gal acini (**Fig 8Ac,e**) displayed GADD153 staining similar to parental MCF10A cells (**Fig 8Aa,b**), the aberrant acini formed by MCF10A cells expressing PERKΔC were largely negative for GADD153 expression (**Fig 8Ad,f and 8B**). The fact that caspase-3 activation was not inhibited to the same extent by PERKΔC, (**Fig 5C and D**) suggests that GADD153 induction might be an event associated with inhibition of proliferation rather than induction of apoptosis.

**Figure 9:**
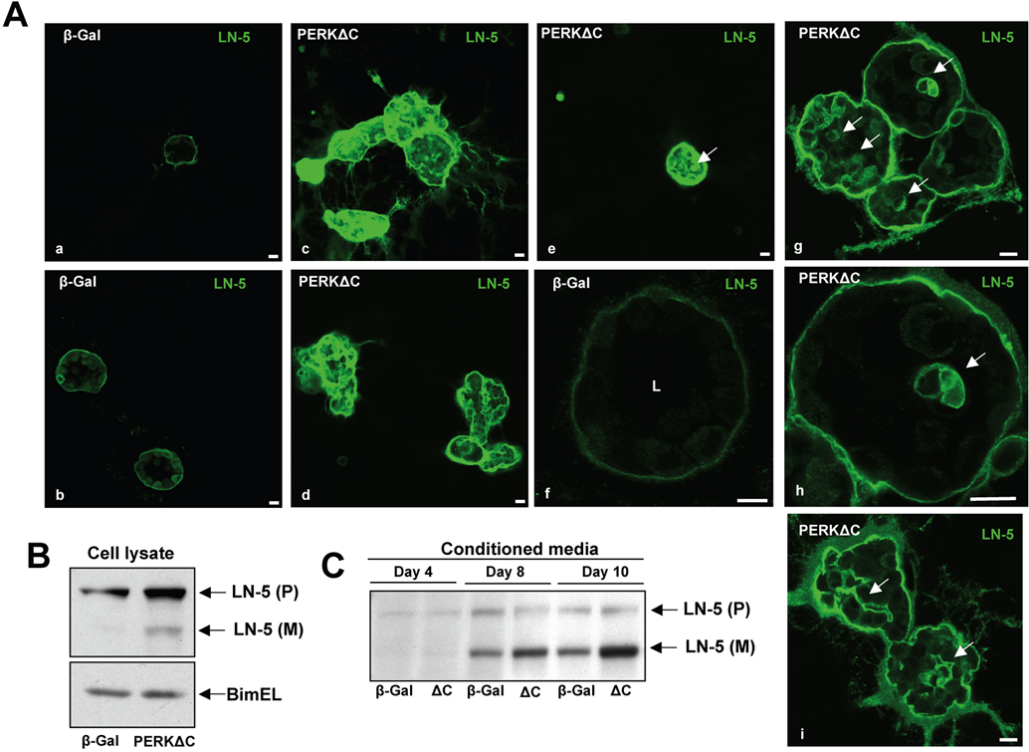
PERK Inhibition Results in Increased Laminin-5 Production, Secretion and Deposition. (A) Confocal images showing the equatorial cross sections through β-Gal (A-a, A-b and A-f) and PERKΔC (A-c to A-e and A-g to A-i) acini stained for Laminin-5 (green), on Day 8 in 3D. Note the increased staining and disorganized Laminin-5 deposition in PERKΔC cells as compared to proper basal localization in the β-Gal control cells. Panels A-e and A-g through A-i (arrows) show details of intra-acinar deposition of Laminin-5 within and around cells in the filled lumen. Scale bars = 10 µm; A-h is a magnified image of an acinus shown in A-g. (B) Western blot analysis from β-Gal and PERKΔC cell lysates shows the precursor (P) and mature (M) forms of LN-5. BimEL was used as loading control. (C) Western blot for Laminin-5 secreted into the conditioned media by β-Gal and PERKΔC cells grown in 3D-Matrigel. For conditioned media, samples corresponding to days 4 and 8, the supernatant was collected after 96 hrs of 3D culture. For the conditioned media sample corresponding to day 10, the supernatant was collected after 48 hrs of 3D culture. ΔC = MCF10A-PERKΔC cells. Note that while in cell lysates (B) the precursor is predominantly detected, in the conditioned media there is stronger detection of the mature form of the Laminin-5-γ2 subunit.

The ability of MCF10A cells to deposit basal Laminin-5 (LN-5) is a characteristic of mammary epithelial cells *in vivo* and allows for proper organization and polarity of the basal epithelial layer [29]. The noticeable disorganization in acini architecture caused by PERK inhibition (**Fig 5** **and** **6**) led us to test whether aberrant deposition of LN-5 might be associated with the ability of cells to grow in the lumen. β-Gal expressing cells showed an outer rim of LN-5 staining that clearly delineated the proper basal location of the BM of the control acini (**Fig 9Aa, b and f**). However, to our surprise PERKΔC acini showed a much stronger staining for LN-5 (**Fig 9Ac, d and e**), suggesting increased expression and/or deposition of LN-5. This suggests a loss of organization since the LN-5 signal was detected around individual cell bodies in the rim and also within the multi-acinar structures (**Fig 9Ae, g, h and i, arrows**). This effect was not dependent on colony size as smaller PERKΔC colonies also showed stronger LN-5 staining within the lumen-filled acini (**Fig 9Ae**). Western blots confirmed that LN5-γ-2 subunit (precursor and mature form) is upregulated in 2D cell lysates of PERKΔC expressing cells (**Fig 9B**). Further, as shown in **Fig 9C**, culture in 3D over time yields more of the mature form of LN-5 detected in the media of PERKΔC vs. β-Gal cells. These results suggest that increased LN-5 production, secretion and deposition around and/or within the amorphous acini may contribute to the strong IF signal. Our data shows that inhibition of PERK can have important consequences on acinar homeostasis as revealed by reduced GADD153 expression and increased expression and enhanced deposition of LN-5.

### Inhibition of PERK Signaling Results in Upregulation of ErbB-1 and ErbB-2 and Tumor Formation *in vivo*

**Figure 10:**
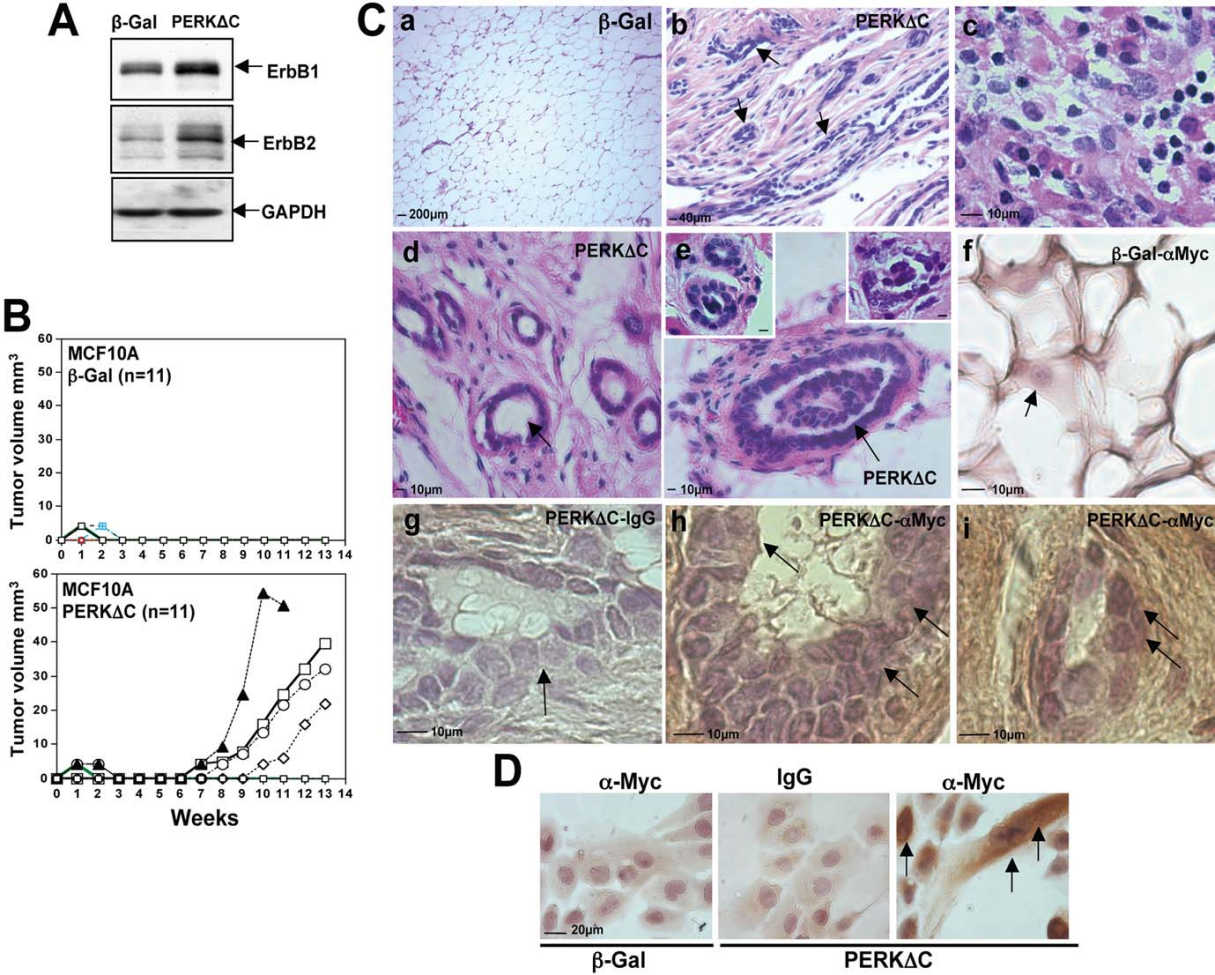
PERK Inhibition in MCF10A Cells Favors Mammary Tumor Formation. (A) Western blot for ErbB1 (EGFR) and ErbB2 (Her2) levels in adhered β-Gal or PERKΔC cells show increased levels when PERK is inhibited. GAPDH was used as loading control. (B) β-Gal or PERKΔC cells were injected orthotopically into the contralateral abdominal mammary fat pads of 3 week-old female nude mice (see methods for details). Post-implantation mice were monitored biweekly for tumor take and when detected tumor diameters were measured and the volume was calculated and plotted as described in methods. Note that none of the mice implanted with β-Gal-MCF10A cells developed tumor nodules. (C) Histology of mammary glands and tumors in mice implanted with β-Gal and PERKΔC expressing MCF10A cells. (C-a) H&E staining of a cleared mammary fat pad inoculated with β-Gal cells. Only adipose tissue and occasional stromal cells that were negative for β-galactosidase activity was observed. (C-b) H&E staining of a PERKΔC tumor lesion. Note the disorganization of the fibrotic-epithelial tissue. Arrows depict the presence of epithelial cells forming acinar or duct-like structures within the PERKΔC tumor lesion. (C-c) Higher magnification of intratumoral accumulations of cells without a defined architecture but comprised of a mixture of epithelial (larger nuclei and pink cytoplasm) and inflammatory cells (smaller darkly stained nuclei). (C-d and e) PERKΔC cells form acini-like structures with an empty lumen or show hyperplastic growth and a repopulated lumen (C-e, top left and right insets). (C-f) Anti-Myc (αMyc) staining in control β-Gal injected mammary fat pads. Note that only a light background signal is observed in adipocytes. (C-g) Histological section of a PERKΔC acinus-like structure stained with a non-specific IgG (arrow denotes the lack of staining in these epithelial cells) or with anti-Myc 9E10 mAb (C-h and C-i); arrows denote the brown staining generated by Myc-tag detection. (D) β-Gal (left panel) or PERKΔC (middle and right panels) cells grown on 2D coverslips and fixed and stained with a non-specific IgG (IgG) or with an anti-Myc 9E10 mAb (α-Myc). The Myc staining was characteristic of intracellular cistern distribution.

The large acinar structures developed by PERKΔC expressing MCF10A cells were to some extent similar to those generated by activation of ErbB2 [2]. In addition, LN-5 is known to favor breast carcinoma progression [45] and the expression of growth factor receptors such as ErbB2 can be regulated at the translational level [46]. Therefore we tested whether ErbB2 expression might be upregulated in PERKΔC vs. β-Gal cells. Western blot analysis revealed that ErbB1 and ErbB2 were upregulated by 2.6 and 2.9 fold in PERKΔC cells, respectively (**Fig 10A**). This suggests that the aberrant behavior of PERKΔC cells might in fact reflect a transformed phenotype. Thus, we tested whether PERKΔC cells might have a proliferative advantage over β-Gal control cells *in vivo*. MCF10A cells are non-tumorigenic in nude mice [47], therefore we performed an orthotopic implantation of parental-, β-gal- and PERKΔC-MCF10A cells (3×10^6^) in the contra lateral mammary fat pads of female nude mice that had the mouse mammary epithelium removed. This was done anticipating that the behavior and organization of MCF10A cells *in vivo* (single- vs. multi-acinar structures) could be followed better in the mammary tissue stroma without interference from the mouse mammary epithelium. Efficiency of this technique was confirmed by histology and revealed that a very small proportion of mouse ducts were present after denuding the mammary fat pad (data not shown). Follow up of these mice revealed no noticeable macroscopic changes in the site of implantation of parental or β-Gal expressing MCF10A cells (**Fig 10B**). In contrast, and to our surprise, ∼40% of mice (n = 30, total of two independent experiments) implanted with PERKΔC expressing cells developed tumor nodules with a variable latency of 7–10 weeks (**Fig 10B**). Injection of equal amounts of PERKΔC cells (n = 20, total of two independent experiments) in the s.c. mouse tissue in the region of the abdominal mammary glands did not produce any tumor nodules at the same times, highlighting the importance of an orthotopic microenvironment (data not shown). Histopathological analysis revealed that β-Gal control cells [47] did not survive in the epithelium-denuded mammary fat pads, as they did in 3D Matrigel cultures. No acini- or duct-like structures could be observed and if any (few remnant from the fat pad clearing), they did not express β-Galactosidase activity (**Fig 10Ca** and data not shown). In striking contrast, MCF10A cells expressing PERKΔC were able to survive in the mammary fat pad for months after inoculation (**Fig 10B–C**). Most importantly, they developed tumors that revealed a histopathology resembling hyperplasia accompanied by strong fibrosis with a mixture of epithelial, stromal and inflammatory cells (**Fig 10Cb–e**). PERKΔC expressing cells formed an assortment of both normal and irregularly organized acini- and duct-like structures (**Fig 10Cb**). In some regions of the lesion an accumulation of epithelial cells along with an inflammatory or fibrotic infiltrate without a defined epithelial architecture was also evident (**Fig 10Cb arrows and Cc**). Several acini-like structures showed a multi-layered arrangement of epithelial cells characteristic of hyperplasia and in some cases although lumen formation was evident (**Fig 10Cd**) we also found structures where the lumen was either non-existent or filled by epithelial cells (**Fig 10Ce and insets**). We next confirmed that these cells expressed the PERKΔC-Myc transgene by performing IHC for anti-Myc (**Fig 10Cg–i**). β-Gal cells in 2D culture displayed low background staining when using anti-Myc Abs that was comparable to IgG background (**Fig 10D**). In contrast, a strong whole-cell signal was observed for PERKΔC cells grown on 2D coverslips, fixed and stained with anti-Myc Abs (**Fig 10D**). No staining was observed for IgG stained in the duct-like structures of PERKΔC tumors (**Fig 10C–g**). In contrast, the acini-like structures in the area of the PERKΔC lesion (**Fig 10C–b**) were positive for Myc, confirming that these were derived from MCF10A-PERKΔC cells (**Fig 10C–h–i**). Only the stromal tissue within the PERKΔC lesions showed a somewhat stronger Myc staining suggesting that MCF10A-PERKΔC cells may possibly have also populated the stromal fibrotic tissue or perhaps undergone trans-differentiation [41] (**Fig 10C–i**). Alternatively, it is possible that the anti-Myc antibody might cross-react to some degree with mouse stromal cells as some cross reactivity with mouse Myc has been reported for the 9E10 mAb [48]. However, we detected no staining whatsoever in stromal or epithelial cells from the few remnant normal mouse ducts away from the lesion but in the same section (data not shown). We conclude that *in vivo* expression of a truncated PERK dominant negative mutant facilitates the development of tumor lesions comprising hyperplasia and fibrosis.

## Discussion

Our results reveal two important and previously unrecognized salient findings: the first is a link between adhesion signaling and PERK-dependent phosphorylation of eIF2α; the second, is the adhesion-dependent role of PERK in inhibiting proliferation and tumor formation both in 3D *in vitro* and *in vivo* animal models, respectively.

Early studies [8], [49] showed that MEFs in suspension displayed translational repression. More recent studies [50] showed that in NIH3T3 fibroblasts, the suspension-induced translation repression correlated with increased P-eIF2α levels. Our results are in accordance with, and further extend these findings by showing that loss of adhesion can regulate eIF2α phosphorylation in a PERK-dependent manner. That PERK is responsible for eIF2α phosphorylation, is supported by our results showing that *PERK*-/- MEFs display reduced phosphorylation of eIF2α as compared to *wt*PERK MEFs placed in suspension. Further, MCF10A cells expressing dominant negative PERK mutants display reduced P-eIF2α, ATF4 and GADD153 levels upon loss of attachment, while other eIF2α kinases, such as GCN2 and PKR are not affected in suspension. Our results also show that higher levels of P-eIF2α and ATF4 could be detected after blocking β1-integrin function, suggesting that ligand-bound β1-integrin might prevent the activation of PERK and downstream signaling. Further studies are required to determine the exact mechanism of the crosstalk between adhesion receptors and PERK. The inhibition of translation in suspension may be due to the fact that phosphorylation of eIF2α at Ser51 results in the sequestration of its GEF, eIF2B preventing eIF2α-GTP hydrolysis [12]. While ATF4, GADD153 and to a lesser extent XBP-1 were upregulated in suspension, other genes commonly regulated during ER stress such as BiP/Grp78, PDI/Erp72, HSP47 were not upregulated. This suggests that suspension-induced stress activates a pathway that only partially overlaps with the repertoire of genes activated, for example, during the unfolded protein response [13].

Importantly, activation or inhibition of PERK-independent pathways appeared to strongly regulate suspension-induced translational repression, as inhibition of PERK was not sufficient to restore protein synthesis and prevent anoikis. Alternatively, this could be due to residual eIF2α phosphorylation, since as little as 10% of eIF2α phosphorylation can cause a strong repression of translation [12], [22]. Another possibility maybe the fact that the eIF4E pathway may still be inhibited in suspension [51], [52]. It is also possible that phosphorylation of PERK and eIF2α in suspension may have other functions not immediately linked to apoptosis (cleaved caspase-3 as a readout) but to other forms of cell death [53]. Moreover, it was interesting to find that PERKΔC enhanced protein synthesis in adhered conditions and prevented DTT-induced translational repression. This suggests that PERK signaling may be required in the adherent cells to inhibit proliferation and PERKΔC-expressing cells might be refractory to these signals during acinus formation. Perhaps, maintenance of LN-5 expression at basal levels is required to prevent hyper-proliferation as only PERKΔC MCF10A cells were surrounded by large LN-5 deposits and positive for Ki67 in the lumen-filled acini. The signals that activate PERK during acinar morphogenesis are unknown but it is possible that more subtle changes in adhesion or in matrix composition that activate PERK may become evident as the acinus reaches its terminal size [4].

Our results showed that the proliferative and tumor suppressive effect of PERK and eIF2α signaling is not due to their ability to induce anoikis in response to loss of adhesion, but due to the inhibition of proliferation in adherent cells. The PERKΔC-induced stimulation of proliferation and tumor initiation was unexpected and raises the question of the mechanisms behind this phenomenon. The PERKΔC-induced phenotype resembled, although not as strongly, that of ErbB2 activation in MCF10A cells [2], [4]. Is it possible that activation of PERK suppresses proliferation and tumorigenesis by inhibiting the rates of translation of proteins involved in growth factor signaling? Insight into this question is provided from studies by Sonenberg and colleagues [22] showing that expression of a dominant negative PKR (DN-PKR) variant or of an eIF2α -Ser51Ala mutant in mouse NIH3T3 cells induced transformation. It was proposed that specific mRNAs that are poorly translated when translation initiation is low or normal become highly translated upon eIF2α dephosphorylation [22]. Several growth factors (i.e. IGF, EGF, PDGF), tyrosine kinases and transcription factors, that would be prone to such regulation were shown to contain long highly structured 5′-UTRs that can inhibit binding of eIF4F or prevent ribosome scanning [12], [54]. In support of this hypothesis, our results show that in PERKΔC cells there is an upregulation of ErbB1 and ErbB2, suggesting that this may be a source of proliferative signaling in PERKΔC cells. Finally, the upregulation of LN-5 in the PERKΔC multi-acinar structures may favor proliferation and survival of the cells. LN-5, β1 and β4-integrin signaling have been shown to promote proliferation, survival and aid in breast cancer progression [24], [44] [45].

Although still unknown in our system, it is also possible that the PERK-dependent post transcriptional regulation of cyclin D1 expression [17] may play a role suppressing proliferation and tumorigenesis. In addition, it will be important to determine whether knock down of GADD153 or ATF4 phenocopy the effects of PERKΔC and PERK-K618A mutants, as GADD153 at least has been shown to have a growth suppressive function [15]. Furthermore, it will be crucial to determine when during acinar morphogenesis PERK becomes activated and to what extent it phosphorylates eIF2α. Then this level of activation can be modeled using the Fv2E-PERK inducible system to determine the effect of unscheduled activation of PERK at different stages during this process.

Evidence from human mammary epithelial cells (HMECs) transfected with various oncogenes also shows that inhibition of eIF2α phosphorylation may be advantageous for tumor cells [55]. For example, as revealed in the Oncomine database HMEC cells transfected with β-Catenin, c-Src, E2F3 and c-Myc show a downregulation of PERK at the transcript level [55]. Interestingly, c-Src and β-Catenin, but not E2F3 and c-Myc transformed HMECs also show a concomitant upregulation of GADD34 transcript, suggesting that discrete oncogenic insults may take advantage of downregulating this growth suppressive pathway. Furthermore, estrogen receptor negative but not positive tumors show downregulation and upregulation of PERK and GADD34 transcripts, respectively (p<0.001) [56]. Additional studies show an inverse correlation between estrogen receptor status and GADD34 expression [55], [57], [58]. These studies suggest that loss of regulation of eIF2α phosphorylation may have consequences for breast cancer progression. Several rare autosomal-recessive mutations in PERK, which cause deletion of the C-terminus or kinases of no or low activity were described in members of inbred families diagnosed with the Wolcott-Rallison syndrome that develop diabetes [59]. However, whether these occur sporadically in other tissues such as breast and whether they have a role in cancer is unknown.

In support of a role for PERK functioning as an inhibitor of tumor formation, PERKΔC expressing MCF10A cells were able to form benign lesions when implanted orthotopically in nude mice. This suggests that PERK signaling may be important in suppressing the early stages of tumor progression in the breast. Interestingly, studies by Bi et al., [60] have shown that although wild type and PERK-/-MEFs that are immortalized with SV40 T-antigen and then transformed with an active Ras-V12 mutant form tumors with similar incidence, those lacking PERK grow, but at a slower rate [60]. A similar result was observed in HT29 colon carcinoma cells expressing a PERKΔC mutant [60]. Mechanistic analysis revealed that this is in part due to in the inability of Ras-transformed PERK-/-MEFs to survive hypoxia and signal to mount an angiogenic response [60]. Our data support an anti-proliferative role for PERK in normal mammary epithelial cells in addition to the pro-survival function it might have in hypoxic regions of Ras-induced fibroblastic tumors [60]. In agreement with the latter we have also found in other model systems that PERK has a pro-survival function [19]. It is possible that depending on the context, like other molecules (e.g., Tiam1 [61] TGF-β [62]), PERK signaling may have different functions during tumor progression. It may operate as a tumor inhibitory pathway during tumor initiation but in other cases it may be co-opted and become an advantageous gene for growing tumor masses to adapt to hypoxic stress [60]. This function has also been attributed to other arms of the UPR [63]. In support of the hypothesis that ER stress signals can be tumor suppressive, recent findings showed that ATF6, ATF4 and XBP-1 signaling can suppress H-Ras induced transformation in melanocytes [21]. Further as mentioned before, eIF2α was found to suppress transformation, since NIH3T3 mouse fibroblasts expressing a Ser-51 to Ala mutant of eIF2α become transformed and tumorigenic *in vivo* [22]. The effects of complete PERK inhibition in normal mammary gland development are not known, since PERK knockout mice display severe neonatal diabetes and die shortly after birth [64]. Thus the generation of PERK conditional knock out mice in the mammary gland epithelium will aid in investigating the function of PERK in this tissue.

To conclude, our studies reveal that PERK activation and possibly downstream eIF2α signaling is regulated by adhesion through an as yet unknown mechanism. This signal appears to be required to limit proliferation and allow for normal acinar morphogenesis. Most importantly, this pathway appears to have a tumor suppressive effect. It is possible that phosphorylation of eIF2α and inhibition of proliferation is a rapid mechanism for mammary epithelial cells to adapt to changes in the microenvironment (i.e. alterations in the stromal compartment, hormonal regulation) or to systemic or environmental factors that could cause damage and subsequent unscheduled or deregulated growth. Thus, loss of PERK signaling might influence downstream genetic and/or epigenetic changes favoring hyper-proliferative disorders characteristic of early steps of breast cancer progression.

## Materials and Methods

### Cell Culture and Materials

Low passage MCF10A cells were maintained as described previously [39], [47]. For 3D cultures, cells were plated on commercially available matrix (growth-factor-reduced Matrigel with protein concentrations between 9–12 mg/ml) from BD Biosciences (San Diego, CA) and purified mouse laminin-1 was obtained from Chemicon International (Temecula, CA). Primary HMECs were obtained from Cambrex (East Rutherford, NJ) and cultured according to manufacturers instructions. HEK293T cells were maintained in DMEM supplemented with 10% FBS and100 U/ml penicillin/streptomycin. Wild type and PERK-/-mouse embryo fibroblast cells (MEFs) were kindly provided by Dr. David Ron (NYU) and maintained as described [14]. AP20187 was obtained from ARIAD Pharmaceuticals, Cambridge, MA (www.ariad.com/regulationkits).

### Antibodies

Rabbit anti-phospho-PERK (16F8), anti-phospho-eIF2α (Ser 51), anti total-eIF2α, anti-phospho and total PKR and GCN2, anti-Bim and anti-cleaved caspase-3 were from Cell Signaling (Danvers, MA). Anti-c-Myc, anti-E-cadherin and anti-GM130 were from BD Biosciences, rabbit anti-phospho-PERK (sc-32577-R), anti-total PERK (H-300), anti-ATF4 and anti-GADD153 from SantaCruz Biotechnology (Santa Cruz, CA) and Biolegend (San Deigo, CA). Anti-Ki-67 from Invitrogen (Carlsbad, CA), anti-GAPDH from Ambion (Austin, TX). Mouse anti-ESA (Clone 323/A3) was from Neomarkers (Fremont, CA). Function-blocking AIIB2 supernatant and concentrate was obtained from Developmental Study Hybridoma Bank (University of Iowa, Ames, IA). Mouse and rabbit anti-IgG, CyQuant nuclear dye and Alexa 488 or 568 conjugated secondary antibodies were from Sigma (St. Louis, MO) and Invitrogen respectively. HRP conjugated anti-mouse IgG and mounting media were from Vector Laboratories (Burlingame, CA) and Invitrogen respectively. Anti-laminin-5 and HRP conjugated anti-rabbit IgG was from Chemicon (Temecula, CA).

### Retroviral Vectors and Stable Cell Lines

pBABEpuro-PERKΔC (lacking the kinase domain of PERK) and pBABE-Fv2E-PERK have been previously described [43]. The Fv2E dimerization kit was from Ariad Pharmaceuticals (www.ariad.com/regulationkits). The *myc*-tagged PERK-(K618A) mutant construct was obtained from J. Alan Diehl (UPenn, PA) and cloned into a pBABEneo retroviral vector. Retroviral vectors were transfected in the Phoenix Ampho retroviral packaging cell line, maintained in DMEM supplemented with 10% FBS and 100U/ml penicillin/streptomycin. All stable cell lines were generated by retroviral infection of MCF10A cells followed by treatment with 2.5 µg/ml puromycin or 400 µg/ml neomycin selection. Stable MCF10A cells expressing the Fv2E-PERK fusion protein were generated, where the kinase domain of PERK is fused to the modified FKBP (Fv) domain, and is activated through dimerization with the bivalent ligand AP20187 in nanomolar concentrations. A pBABEpuro-βGalactosidase construct expressing β-Galactosidase served as vector control.

### Anoikis Assays

Tissue culture plates were coated with sterile 1% agar dissolved in PBS and allowed to solidify before use. MCF10A, primary HMECs, and HEK293T cells were placed in the appropriate growth medium containing serum, in the presence of 0.5% methylcellulose (to avoid clumping of cells) in suspension and plated on agar-coated dishes in a humidified 37°C incubator. For Fv2E-PERK stable cell lines, cells were treated with 2nM of AP20187 (added fresh daily) or equal volume of EtOH as control. Integrin function blocking antibody experiments were carried as follows: Laminin-I (10 µg/ml) in PBS was coated on plates overnight at 37°C, washed with PBS and blocked with 0.1mg/ml BSA at 37°C for 1 h. For suspension, cells were plated on 1% agar in 0.5% methylcellulose as above. MCF10A cells were detached with 2mM EDTA in PBS, resuspended in serum-free DMEM:F12 and equal numbers of cells (1–2.5×10^6^) were pre-incubated with 10 µg/ml AIIB2 or 10 µg/ml control IgG for 30 min at 37°C, prior to plating in media containing 1% serum with all supplements for an additional 24h.

### Immunoblotting

Cells from either adhered or suspended cultures were washed with ice cold PBS, lysed with RIPA buffer containing protease and phosphatase inhibitors and processed for immunoblot analysis as described previously [19]. For detection of phospho- and total PERK in MCF10A cells, 100 µM β-glycerophosphate was added to cells 30 min prior to lysis in an attempt to preserve PERK phosphorylation. Adhered or suspended cells were washed with ice cold PBS followed by PBS-EDTA, and lysed on ice with Lysis buffer containing 1% Triton-X-100, 150 mM NaCl, 20 mM Hepes, 10% glycerol, and 1 mM EDTA. Sodium orthovanadate (1 mM), 100 mM NaF and 17.5 mM **β**-glycerophosphate, 1mM PMSF, 4 µg/ml Aprotinin and 2 µg/ml Pepstatin A were added freshly before use [14]. 20 µg of total protein was separated on a 6% SDS polyacrylamide gel. PERK antibodies were used at a dilution of 1∶200.

### RT-PCR Analysis

ATF4, GADD153, XBP-1, BiP, HSP47 and PDI/Erp72 mRNA was analyzed using 1-2 µg of total RNA isolated from MCF10A, or HEK293T cells (Trizol reagent, Invitrogen) grown adhered or in suspension as described above using the Retroscript two-step RT-PCR kit from Ambion according to manufacturer's instructions. GAPDH was used as loading control. Primer sequences used were: GADD153 (F) 5′-GGAAGCCTGGTATGA-GGACC; GADD153 (R) 3′-CCAATTGTTCATGCTTGGTG, ATF4 (F) 5′-TCAAACTTCA-TGGGTTCTCC-3′; ATF4(R) 5′-GTGTCATCCAACGTGGTCGTCAG-3′, XBP-1 (F) 5′-CCTTGTAGTTGAGAACCAGG-3′; XBP-1 (R) 5′-GGGGCTTGGTATATATGTGG-3′, BiP (F) 5′-GGGTGGCGGAACCTTCGATGTGTC-3′; BiP (R) 5′-ATTTGGCCCGAGTCA-GGGTCTCAG-3′, Hsp47 (F) 5′-TGCAGTCCATCAACGAGTGGGCCG-3′; Hsp47 (R) 5′-TCGTCGTCGTAGTAGTTGTAGAGGC-3′, PDI/Erp72 (F) 5′-CAATACCAGGATGCC-GCTAACAACC-3′; PDI/Erp72 (R) 5′-GCATCGTTTGACACCTTGCGGTGG-3′ and GAPDH (F) 5′-CGTCATGGGTGTGAACCATGAG-3′; GAPDH (R) 5′-GTAGACGGCAGGTC-AGGTCCA-3′.

### Transient cDNA Transfections

Cells were transiently transfected using FuGene™ transfection reagent (Roche) with a pGADD153-EGFP reporter (a gift from Dr. S. Howell, UCSD) alone or with a pFLAG-CMV-2-GADD34 or a pcDNA3.1Hygro plasmid. 24 h post-transfection, cells were either left adhered or detached by mild trypsinization and put into suspension on 1% agar coated tissue culture plates with 0.5% methylcellulose for an additional 24 or 48 hours, and analyzed by flow cytometry as described [19].

### FACS Analysis

GFP fluorescence was quantitated using Epics ALTRA (Beckmann Coulter) or LSRII (BD Pharmingen) flow cytometers as described [19]. For anoikis assays, 10,000 events were collected and the percentage of apoptotic cells in adhered or suspension cultures was detected using propidium iodide staining (BD Pharmingen). The sub-G0 cell population was gated as the apoptotic fraction. Unstained cells served as negative control.

### Metabolic Labeling and Polysome Gradients

To measure protein synthesis, 0.5×10^6^ MCF10A cells were seeded in 60 mm dishes both in adhered or in suspension conditions as described above. 24 hrs later the cells were washed twice with PBS and incubated with methionine/cysteine- free DMEM:F12 media containing 10% HS for 1h. Cells were then pulse-labeled for 30 min with medium containing 50 µCi/mL of Redivue Pro-mix L-[^35^S] cell labeling mix (GE Healthcare, Piscataway, NJ) and lysed as described [19]. 5 µl of lysate/lane was loaded onto a 10% gel and analyzed by Coomassie staining for protein loading and by autoradiography for the detection of ^35^S-methionine incorporation. For polysome gradient analysis MCF10A cells (6×10^6^ cells/15 cm plate) were plated at 50% confluence in adhered or suspension conditions 24 hrs before analysis and analyzed as described previously [14].

### Mice and Transplantation of Cleared Mammary Fat Pads

Female FVB nude mice were anesthetized with ketamine, given i.p. in a mixture containing 1 ml ketamine, 1 ml xylazine and 4.2 ml sterile saline (dose of 0.1 ml/30 g body weight) for 40–45 min. The abdominal (4^th^) mammary glands of 3-week-old female mice were cleared of endogenous epithelium by surgical excision just distal of the mammary lymph node, a positional landmark. On day 0, β-Gal control or PERKΔC-expressing MCF10A cells were suspended in serum free media in a 50-µl volume (3×10^6^ cells/mouse) and injected in the cleared fat pad using a 25 gauge needle to avoid damaging the cells. Mice were injected with xylazine for two days post-surgery. Mammary glands were palpated twice a week to detect any growth at the inoculation sites. Tumor volume in the PERKΔC-transplanted mammary fat pads was measured using calipers and calculated according to the following equation: (Length×width^2^)/2 = tumor volume (mm^3^). The glands with or without tumors were dissected and processed for sectioning or stained with hematoxylin-eosin. Briefly, mounted glands were left for 2–3 mins at RT to adhere to glass slides, then transferred to 10% formalin (Sigma) and fixed overnight at 4°C. The next day, samples were washed twice with 70% ethanol, detached from the slides, placed between sponges inside histology cassettes submerged in 70% ethanol and sent to the Histology Core facility (MSSM, NY) to be dehydrated and processed for paraffin embedment. All animal protocols were approved by the IACUC committee, MSSM, NY and SUNY-Albany, NY.

### Immunohistochemistry for c-Myc Expression

Sections from control β-Gal or PERKΔC transplanted mammary fat pads were fixed and processed for c-Myc staining. Briefly, tissues were deparaffinized in xylene followed by three washes in a graded series of ethanol, then permeabilized for 10 minutes in 0.5% Triton-X 100. Slides were rinsed with PBS, dehydrated in a graded series of ethanol, and dipped in 3% hydrogen peroxide and methanol for 20 minutes to block endogenous peroxidase. Slides were then rehydrated, blocked for 1 hr in PBS plus normal goat serum, following vendor instructions (Vector Laboratories, Vectastain Elite ABC Kit) and incubated for 10 minutes at room temperature with either an anti-C-myc antibody (BD Biosciences) or a control IgG (Sigma). Slides were incubated with a biotinylated secondary antibody (Vectastain Elite ABC Kit) for 1 hour at RT and antibody binding was detected with the Vectastain ABC Kit as indicated by the vendor. The peroxidase activity was developed by diaminobenzidine and nuclei were counterstained with Hematoxilin.

### Immunofluorescence and Confocal Microscopy

Briefly, for detection of GADD153 and ATF4 in adherent vs. suspended cells these were either grown on glass coverslips (adherent) or collected by centrifugation from suspension cultures and allowed to attach to polylysine-coated coverslips for 30 min and fixed with 3% paraformaldehyde and stained following standard protocols [19]. Images were captured using a Nikon Eclipse TS100 epifluorescence microscope fitted with a digital SPOT-RT camera. The protocol for indirect IF of MCF10A cells in 3D has been previously described [39]. Confocal images were acquired by using a Leica TCS SP5 inverted confocal microscope (Leica Microsystems, USA) with a 40× objective or with a LSM5 Meta (Zeiss) with 25× objectives. Raw images were visualized and analyzed using Adobe Photoshop 6.0, SPOT and ImageJ software.
